# Transitions between frailty states in the very old: the influence of socioeconomic status and multi-morbidity in the Newcastle 85+ cohort study

**DOI:** 10.1093/ageing/afaa054

**Published:** 2020-04-28

**Authors:** Nuno Mendonça, Andrew Kingston, Mohammad Yadegarfar, Helen Hanson, Rachel Duncan, Carol Jagger, Louise Robinson

**Affiliations:** 1 Population Health Sciences Institute, Newcastle University, NE2 4AX, UK; 2 EpiDoC Unit, CHRC, NOVA Medical School, Universidade Nova de Lisboa (NMS-UNL), Lisbon, Portugal; 3 Leeds Institute of Cardiovascular and Metabolic Medicine, Leeds University, UK; 4 The Newcastle upon Tyne Hospitals NHS Foundation Trust, NE1 4LP, UK

**Keywords:** *older people, education*, *deprivation*, *frailty*, *aged 80 and over*, *multi-morbidity*

## Abstract

**Background:**

Using Newcastle 85+ Study data, we investigated transitions between frailty states from age 85 to 90 years and whether multi-morbidities and socioeconomic status (SES) modify transitions.

**Methods:**

The Newcastle 85+ Study is a prospective, longitudinal cohort study of all people born in 1921 in Newcastle and North Tyneside. Data included: a multidimensional health assessment; general practice record review (GPRR) and date of death. Using the Fried phenotype (participants defined as robust, pre-frail or frail), frailty was measured at baseline, 18, 36 and 60 months.

**Results:**

Frailty scores were available for 82% (696/845) of participants at baseline. The prevalence of frailty was higher in women (29.7%, 123/414) than men (17.7%, 50/282) at baseline and all subsequent time points. Of those robust at baseline, 44.6% (50/112) remained robust at 18 months and 28% (14/50) at age 90. Most (52%) remained in the same state across consecutive interviews; only 6% of the transitions were recovery (from pre-frail to robust or frail to pre-frail), and none were from frail to robust. Four or more diseases inferred a greater likelihood of progression from robust to pre-frail even after adjustment for SES. SES did not influence the likelihood of moving from one frailty state to another.

**Conclusions:**

Almost half the time between age 85 and 90, on average, was spent in a pre-frail state; multi-morbidity increased the chance of progression from robust and to frail; greater clinical intervention at the onset of a first chronic illness, to prevent transition to multi-morbidity, should be encouraged.

## Key Points

- This is the first study to report frailty progression in the very old at four time points over 5 years.
- On average, around half the time between age 85 and 90 are spent pre-frail and just under 1 year frail.
- Between age 85 and 90, most remained in the same frailty state or progressed to adjacent states.
- Progression from robust to pre-frail, and from pre-frail to frail, is more likely with four or more diseases.
- Socioeconomic status did not appear to have an effect on the transition from one frailty state to another.

## Introduction

Frailty has been defined as an increased vulnerability to poor health stressors due to a state of increased vulnerability to poor resolution of homoeostasis after a stressor event [1]. In the very old, those older than 85 years, between a quarter to a half of people are estimated to be frail bringing an increased risk of falls, hospitalisation, care home admission and death; however accurate figures are difficult to achieve as the concept and diagnostic criteria of frailty vary [1, 2]. In research, two frailty models are in common use, the frailty phenotype [3] and Rockwood’s cumulative deficits model [4]. Both have developed scales/tools to assess frailty in practice, the Fried Frailty Index and the Rockwood Frailty Index respectively. The Fried frailty index (FFI) categorises individuals into three frailty states: robust, pre-frail or frail. This FFI examines changes in the body systems of individuals and is constructed to reflect impairment that leads to an overall manifestation of a more vulnerable state. Specifically, these pertain to weight loss, exhaustion, slowness and low levels of physical activity. The RFI approaches the concept from a more disease-centric perspective. It counts the number of ‘health deficits’ observed (or diagnosed) for individuals, and the greater the number of health deficits, the greater the perceived level of vulnerability (and the therefore frailty). The cumulative deficits model has been modified and adapted into an electronic frailty index currently used in primary care in England (eFI) [5]. Additional frailty tools continue to be developed [6].

Frailty is a dynamic state with people moving between robust, pre-frail and frail states [7]; cross-sectional studies have reported that factors such as comorbidities and low socioeconomic status (SES) influence transitions between states. However, few longitudinal studies have assessed the relationship between multi-morbidity and frailty over a significant length of time (i.e. >2 years), with conflicting results reported [8, 9]. The relationship between SES, typically measured by education or income, and frailty trajectories over time has been widely researched internationally [10, 11]. Generally lower SES is associated with a higher chance of worsening frailty [9, 10, 12] and lower chance of recovery. Other studies find no association [13–15], perhaps due to age, since associations appear to be present in young–old age groups rather than the very old [16].

Despite the rapid increases in the very old, there is little research on frailty progression in this important group [7] and specifically if, and how, factors, such as comorbidities or SES, contribute to frailty progression. Also, earlier studies often assess frailty at only two time points and fail to include people in institutional care thereby resulting in an incomplete picture. We aim to address these gaps using data from the Newcastle 85+ cohort [17]; between ages 85 and 90 years, we investigate (i) the progression of frailty over 5 years as measured by the Fried phenotype and (ii) the effect of multi-morbidity and SES on frailty progression, using transitions between frailty states and frailty states and death.

## Methods

### Newcastle 85+ study

A detailed description of the Newcastle 85+ Study, a prospective, observational longitudinal cohort study that commenced recruitment in 2006, has been published [17]. All people born in 1921, permanently registered with a General Practitioner in Newcastle and North Tyneside, were approached. Participants were 85 years old at baseline with follow-up at 18, 36 and 60 months. A recruitment and retention flowchart of the study is presented in Appendix 6. Data was collected using three methods: (i) a multidimensional health assessment comprising questionnaires, measurement and function tests and blood tests, (ii) general practice record review (GPRR) and (iii) mortality data obtained through NHS Digital.

This study was conducted according to the guidelines set out in the 1964 Declaration of Helsinki, and the Newcastle and North Tyneside local research ethics committee approved all procedures involving human subjects (06/Q0905/2). Written informed consent was obtained from all participants, and when that was not possible, consent was obtained from a caregiver or a relative according to the UK Mental Capacity Act 2005.

### Frailty

As we wish to examine the effect of multi-morbidity on transitions to frailty, we used the FFI. This index measures the phenotype of frailty through dysregulated body systems. We could not use the RFI as this would have led to problems with multicollinearity and a self-fulfilling prediction due to the similarities in the constructs of multi-morbidity and RFI. The Fried frailty status (FFS) was derived for each time point based on approximations from the Cardiovascular Health Study (CHS) methodology [3, 17] (Appendix 7). Briefly, FFS was derived by scoring (1) for every component that was present (shrinking, physical endurance/energy, low physical activity, weakness and slow walking speed) and (0) if absent (range 0–5). Participants with a score of zero were defined as robust, with 1–2 as pre-frail and with ≥3 components as frail. The analytic sample consisted of 696 participants with assigned FFS at baseline (no exclusions). The missing pattern analysis of the possible missing items to derive the FFS is included in Appendix 1.

### Socioeconomic status

Full-time education was defined in three categories: up to 9 years, 10–11 years or 12 or more years in full-time education (missing *n* = 2); Participants’ postcodes were used to derive the index of multiple deprivation (IMD) with higher scores representing those living in less deprived areas (missing *n* = 0) [18].

### Mortality and disease count

The time to death was calculated as the time between age at baseline (2006–2007) and time of death (censored at 29 August 2012). Mortality follow-up was restricted to 5 years to match the end of data collection. Disease count was created by scoring eight chronic diseases as either present (1) or absent (0) (cardiac, respiratory and cerebrovascular disease, arthritis, hypertension, diabetes mellitus, cognitive impairment and cancer in the past 5 years) from GPRR at baseline, 18, 36 and at 60 months of follow-up [19].

### Other variables

A disability score was calculated using items predominantly from the Groningen Activity Restriction Scale as previously described [19]. Each activity of daily living and mobility item that the participant could not perform or perform with difficulty was scored as one and with a score of zero if the activities were performed without any difficulty. This formed the disability score and was calculated for baseline and at 18, 36 and at 60 months of follow-up [20]. Global cognition was assessed with the Standardised Mini-Mental State Examination (SMMSE) and depression was assessed by the 15-item Geriatric Depression Scale (GDS) with previously used cutpoints [17]. These were part of the model building strategy but not included in the final model and are used to characterise the sample.

### Statistical analysis

Normality was assessed with Q-Q plots. Categorical data are presented as percentages (with sample size) and non-normally distributed variables as medians and interquartile ranges (IQR). To determine the contribution of socioeconomic inequalities and multi-morbidity to transitions between frailty states (robust, pre-frail and frail) and to death (absorbing state) over 5 years, we used a Markov multistate model with the Broyden–Fletcher–Goldfarb–Shanno (BFGS) algorithm to maximise the likelihood. The allowed transitions in the frailty–death model are shown in Appendix 8. Initially we investigated models individually for education, IMD, and disease count, adjusted for age and sex. We then fitted a full model adjusted for all variables (age, sex, education, IMD, disease count) to see the residual effect of each of the SES measures and disease count on transitions. These variables were selected based on their theoretical, clinical and statistical relevance to a stable parsimonious model. As a sensitivity analysis, missing FFS was inputted based on the existing FFS components, sex, institutionalised or not and MMSE, chronic disease and disability categories. Results are presented as hazard ratios (HR) and 95% confidence intervals (CI). Total length of stay in each of the frailty states was also presented. Point estimates and CI were used to assess statistical and clinical significance. Data management and analysis of baseline characteristics were conducted using Stata v12.0, and multistate models were fitted with the msm package [21] in R v3.5.1.

## Results

FFS could be calculated for 696 participants at baseline (414 women and 282 men). More participants without the necessary variables to assign an FFS state were women, lived in institutions, were less cognitively intact, had more chronic diseases, were more disabled and had shorter survival time than those without FFS (Appendix 2).

### Baseline health and SES characteristics by FFS

At baseline, more women were frail (71%). Compared to those robust, frail participants had poorer self-rating of health (24% of frail *vs* 61% of robust rating their health as excellent or very good), were less cognitively intact (63% of frail *vs* 91% of robust had an SMMSE score ≥ 26), were more depressed (58% of frail *vs* 91% of robust did not have depression), were more disabled (frail participants had a median of eight difficulties with activities of daily living), had more urinary incontinence (50% of frail *vs* 13% of robust), were more visually impaired (42% of frail *vs* 26% of robust) and experienced more falls (26% of frail *vs* 11% of robust). At baseline, more participants living in more deprived areas (Q1) were robust (34.9%) than frail (20.8%), and conversely those living in less deprived areas (Q4) were less robust (20.2%) than frail (32.9%). There was a 10% difference between the number of people with 0–9 years of full-time education that were robust (60.5%) and frail (71.7%) (Table 1).

**Table 1:** Baseline health and sociodemographic characteristics of participants, by frailty state

	Robust (*n* = 129)	Pre-frail (*n* = 394)	Frail (*n* = 173)	All (*n* = 696)
**Women**	47.3 (61)	58.4 (230)	71.1 (123)	59.5 (414)
**Living status**				
Alone	62.0 (80)	57.1 (225)	57.0 (98)	58.0 (403)
Not alone	38.0 (49)	40.9 (161)	34.9 (60)	38.9 (270)
Institution	0.0 (0)	2.0 (8)	8.1 (14)	3.2 (22)
**Self-rated health**				
Excellent/very Good	61.2 (79)	43.6 (170)	23.7 (41)	41.9 (290)
Good	31.8 (41)	38.2 (149)	37.0 (64)	36.7 (254)
Fair/poor	7.0 (9)	18.2 (71)	39.3 (68)	21.4 (148)
**Education**				
0–9 years	60.5 (78)	62.5 (245)	71.7 (124)	64.4 (447)
10–11 years	25.6 (33)	22.7 (89)	20.8 (36)	22.8 (158)
12+ years	14.0 (18)	14.8 (58)	7.5 (13)	12.8 (89)
**IMD**				
More deprived (Q1)	34.9 (45)	27.4 (108)	20.8 (36)	27.2 (189)
Q2 + Q3	45.0 (58)	52.3 (206)	46.2 (80)	49.4 (344)
Less deprived (Q4)	20.2 (26)	20.3 (80)	32.9 (57)	23.4 (163)
**SMMSE**				
Normal (26–30)	90.7 (117)	81.0 (319)	62.8 (108)	78.3 (544)
Mild (22–25)	6.2 (8)	14.5 (57)	21.5 (37)	14.7 (102)
Mod (18–21)	3.1 (4)	3.1 (12)	9.3 (16)	4.6 (32)
Severe (0–17)	0.0 (0)	1.5 (6)	6.4 (11)	2.5 (17)
**GDS** ^a^				
No depression	96.0 (120)	85.2 (323)	58.1 (90)	80.9 (533)
Mild	3.2 (4)	10.6 (40)	21.9 (34)	11.8 (78)
Severe	0.8 (1)	3.2 (12)	16.8 (26)	5.9 (39)
**Disability score** ^b^	1 (0–2)	2 (1–5)	8 (5–11)	3 (1–6)
**Chronic diseases**				
0–1	35.7 (46)	31.7 (125)	14.5 (25)	28.2 (196)
2–3	55.8 (72)	54.3 (214)	54.9 (95)	54.7 (381)
4+	8.5 (11)	14.0 (55)	30.6 (53)	17.1 (119)
**Urinary incontinence**	13.4 (17)	25.5 (100)	50.0 (86)	29.3 (203)
**Impaired vision**	26.4 (34)	35.2 (138)	42.1 (72)	35.3 (244)
**Impaired hearing**	58.9 (76)	57.3 (225)	64.2 (111)	59.3 (412)
**Falls** ^c^	10.9 (14)	15.6 (61)	26.0 (45)	17.3 (120)

^a^Forty-six participants had SMMSE<15 and were not assessed.

^b^Median (interquartile range).

^c^Defined as at least one fall in the previous 12 months.

Entries are percentages (%) and counts (*n*) unless otherwise stated. GDS, Geriatric depression scale; SMMSE, Standardised Mini-Mental State Examination; Mod, moderate.

### Frailty states at baseline, 18, 36 and 60 months

**Figure 1:**
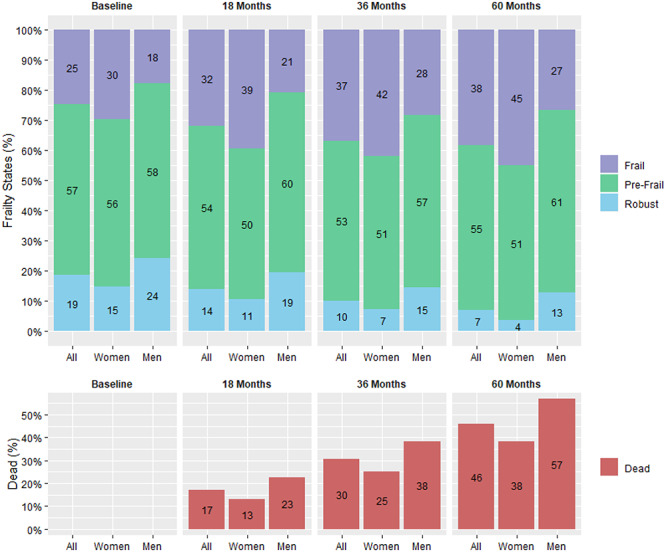
Fried frailty states and death (%), by follow-up and sex.

Figure 1 and Appendix 3 show a progressive decrease in robust FFS (19% of all participants at baseline and 7% of surviving participants at 60 months) and increase in frail FFS (25% of all participants at baseline and 38% at 60 months) in men and women over the 5 years of follow-up.

### Transitions between FFS and to death from age 85 to 90 years

At consecutive intervals most participants (52%) remained in the same state over the 5 years. Thirty percent of the transitions were to adjacent states (pre-frail, frail or death). Only 6% of the transitions were recovery (i.e. from pre-frail to robust or frail to pre-frail), and none were from frail to robust (Appendix 4). On average an 85-year-old can expect to spend 1.44 (95%CI: 1.17–1.74) years robust, 2.21 (95%CI: 2.01–2.36) years pre-frail and 0.80 (95%CI: 0.68–0.93) years frail over the next 5 years.

### Effect of socioeconomic inequalities on transitions between FFS from 85 to 90 years

**Figure 2:**
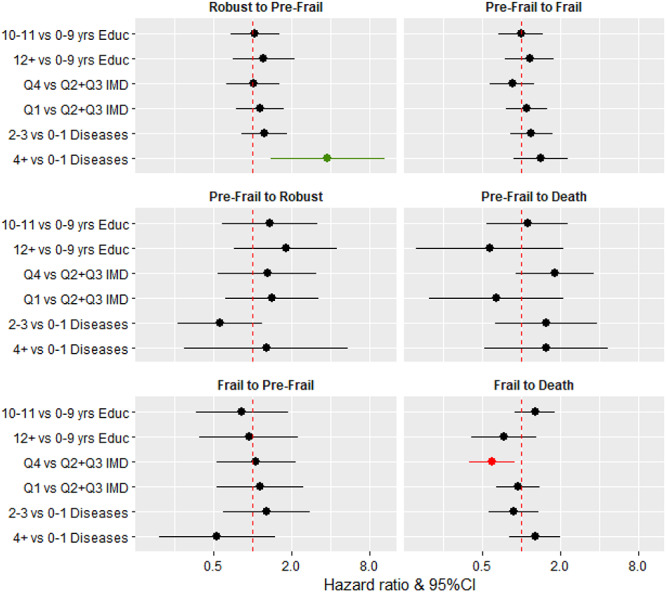
Hazard ratios and 95% confidence intervals for socioeconomic inequalities, disease count (multi-morbidity) and transitions between frailty states and death. Educ, years of full-time education; IMD, index of multiple deprivation. All three separate models (education, IMD and disease count) were adjusted for age and sex. Quartiles of IMD were as follows: Q1 (25th percentile), 3–12; Q2 + Q3 (25–75th percentile), 12–43; Q4 (75th percentile), 43–78.

The likelihood of transitioning between frailty states and to death was similar between groups defined by education in models adjusted for age and sex (Figure 2) and in models adjusted for IMD and number of chronic diseases (Appendix 5). Those living in less deprived areas (higher IMD) were less likely to die from a frail state (HR: 0.60, 95%CI: 0.40–0.89) in models adjusted for age and sex, and this remained after further adjustment for education and disease count (HR: 0.59, 95%CI: 0.39–0.90) (Appendix 5). Multiple imputations of the missing FFS did not alter the results significantly except for the transition from pre-frail to robust where those in the highest quartile of IMD were less likely to recover (HR: 0.46, 95%CI: 0.22–0.95), likely an effect of imputing missing robust FFS.

### Effect of multi-morbidity on transitions between FFS from 85 to 90 years

Participants with four or more diseases were more likely to transition between a robust and pre-frailty state than those with zero or one diseases in models adjusted for age and sex (HR: 3.78, 95%CI: 1.38–10.38) (Figure 2) and further adjusted for education and IMD (HR: 3.79, 95%CI: 1.48–9.75) (Appendix 5). The trend was similar for the transition between pre-frail and frailty (HR: 1.65, 95%CI: 1.06–2.57 Appendix 5).

## Discussion

This is the first study to report frailty progression in very old people at four time points from age 85–90 years and specifically to investigate the role of multi-morbidity and SES on frailty progression. Our findings show that between age 85 and 90, on average just under half the time (2.2 years) is spent pre-frail and around 9 months (0.8 years) frail. Most very old adults remained in the same frailty state or progressed to adjacent states (pre-frail, frail or death), between age 85 and 90, with recovery from pre-frail or frail accounting for only 6% of transitions. Compared to having none or one chronic disease, four or more diseases inferred a greater likelihood of progression from robust to pre-frail and from pre-frail to frail. However, SES did not appear to have an effect on the likelihood of moving from one frailty state to another at this age.

As reported in the literature, our baseline findings show that those defined as frail were more likely to be female [7, 9, 10] and have depression or cognitive impairment. However comparison of our results on frailty progression with previous research is difficult because other studies measuring frailty at four or more time points have only included younger old populations [11, 16, 22], who theoretically may be healthier. For example, in a recent systematic review exploring frailty transitions in community-dwelling older people (16 studies; 42,775 participants) the mean age of the participants ranged from 69 to 78 years [7]. As in this review and other studies [13], most of the transitions in our very old cohort were in the forward direction (e.g. robust to frail), with only a very small minority transitioning from frail to robust as per in younger old cohorts [7].

With respect to SES, some prior longitudinal, observational studies have reported an effect on frailty transitions [9, 10, 12, 23], whilst others have not [13–15]. In our study, participants living in more affluent areas (top 25% of IMD) were less likely to die from a frail state; there was also a non-significant trend for this group to be more likely to die from a pre-frail state and be less likely to transit from pre-frail to frail. This may mean that very old adults living in an area with a higher IMD score (late-life SES indicator) spend their final years pre-frail rather than frail. However, it might also be that very old, frail individuals in more affluent areas live longer in a frail state through better care. Education appeared to have little effect on the likelihood of transitioning between frailty states or to death, and this is consistent with others [16], suggesting that education (early-life SES) is less important in very old age than current SES (IMD), although we have found the converse to be true for disability transitions in this population [19].

A potential reason for the paucity of evidence of associations with SES pertains to survivor bias. As our population are all aged 85 at baseline, those of poorer health and from lower SES may already have died. Furthermore, those who recruited to the study from low SES may exhibit more robust health than their decedent counterparts, despite low SES, thus diluting effects observed in the younger old.

In terms of multi-morbidity, our participants with four or more diseases were more likely to move from robust to pre-frail or from pre-frail to frail. This is similar to a Chinese study [9] but contrasts with a German study where there was no association between comorbidity and frailty over 1.5 years in a population of 1,600 adults aged 80+ years. In terms of clinical care to reduce or delay the onset of frailty, intervention at the onset of the first chronic illness to prevent the onset of multi-morbidity may be appropriate.

Major strengths of our study include the use of GP records at every phase to determine the number of chronic diseases and the all-inclusive recruitment strategy (only people excluded from the study were those with end-stage terminal illness). Unfortunately, we could not assign a frailty score at baseline for 149 participants (17.6%); it is therefore possible that those not included in our study were frailer than the participants who could be classified. However classifiable participants had complete follow-up data at 18, 36 and 60 months, including mortality data, and so we expect the impact to be minimal. With observations at 18, 36 and at 60 months, it is feasible that we may have missed transitions to some frailty states potentially leading to underestimation of the effect of SES.

We hypothesise two reasons for failing to find evidence of an effect of multi-morbidity on transitions from frail states to death: (i) multi-morbidity in this age group has a greater effect on mortality than frailty incidence (as observed), and/or (ii) multi-morbidity does impact frailty in this age group, but it results in a swift transition to death, and due to the time-intervals between the interview schedules, we miss transitions to frailty.

Frailty has been identified as a common condition associated with death in community-dwelling older people [5] and is potentially a method of predicting those most at risk of dying, especially in the last year of life [1, 24]. However recent research showed that whilst the eFI is a strong predictor of mortality at the population level, it is not clinically helpful having a low predictive value for mortality at an individual level, even close to death [25]. Frailty can be ‘improved’ [26], with a recent review suggesting the easiest and most effective intervention is a combination of strength exercises and protein supplements [27]. Unfortunately accurately ‘diagnosing’ frailty and its various states remains a clinical challenge, hampered by a lack of consensus over the definition and conceptualisation of frailty, with growing numbers of frailty assessment tools [1, 6, 28]. As we found, around half of those robust at baseline became pre-frail by 18 months and only 6% frail; future research may be better targeted at identifying the pre-frail than the already frail or as recently suggested reversing the current negative clinical paradigm and focusing on what an older person can achieve rather than what they cannot do [29, 30].

## Supplementary Material

File002_afaa054

## Data sharing statement

Newcastle 85+ Study data may be obtained by agreement from the Data Guardians Group. Full guidance is available on the Newcastle 85+ Study website which can be accessed at https://research.ncl.ac.uk/85plus/

## References

[1] Clegg A, Young J, Iliffe S, Rikkert MO, Rockwood K Frailty in elderly people. The Lancet 2013; 381: 752–62. 10.1016/S0140-6736(12)62167-9 PMC4098658 23395245

[2] Song X, Mitnitski A, Rockwood K Prevalence and 10-year outcomes of frailty in older adults in relation to deficit accumulation. J Am Geriatr Soc 2010; 58: 681–7. 20345864 10.1111/j.1532-5415.2010.02764.x

[3] Fried LP, Tangen CM, Walston J et al. Frailty in older adults: evidence for a phenotype. J Gerontol A Biol Sci Med Sci 2001; 56: M146–57. 11253156 10.1093/gerona/56.3.m146

[4] Rockwood K, Song X, MacKnight C et al. A global clinical measure of fitness and frailty in elderly people. Can Med Assoc J 2005; 173: 489–95. 16129869 10.1503/cmaj.050051 PMC1188185

[5] Clegg A, Bates C, Young J et al. Development and validation of an electronic frailty index using routine primary care electronic health record data. Age Ageing 2016; 45: 353–60. 26944937 10.1093/ageing/afw039 PMC4846793

[6] Buta BJ, Walston JD, Godino JG et al. Frailty assessment instruments: systematic characterization of the uses and contexts of highly-cited instruments. Ageing Res Rev 2016; 26: 53–61. 26674984 10.1016/j.arr.2015.12.003 PMC4806795

[7] Kojima G, Taniguchi Y, Iliffe S, Jivraj S, Walters K Transitions between frailty states among community-dwelling older people: a systematic review and meta-analysis. Ageing Res Rev 2019; 50: 81–8. 30659942 10.1016/j.arr.2019.01.010

[8] Vetrano DL, Palmer K, Marengoni A et al. Frailty and multimorbidity: a systematic review and meta-analysis. J Gerontol: Series A 2018; 74: 659–66. 10.1093/gerona/gly110 29726918

[9] Zheng Z, Guan S, Ding H al E. prevalence and incidence of frailty in community-dwelling older people: Beijing longitudinal study of aging II. J Am Geriatr Soc 2016; 64: 1281–6. 27321607 10.1111/jgs.14135

[10] Etman A, Burdorf A, Cammen TJM V der, Mackenbach JP, Van Lenthe FJ Socio-demographic determinants of worsening in frailty among community-dwelling older people in 11 European countries. J Epidemiol Community Health 2012; 66. 10.1136/jech-2011-200027 22544921

[11] Hoogendijk EO, Heymans MW, Deeg DJH, Huisman M Socioeconomic inequalities in frailty among older adults: results from a 10-year longitudinal study in the Netherlands. Gerontology 2018; 64: 157–64. 29055946 10.1159/000481943 PMC5841137

[12] Wang C, Song X, Mitnitski A et al. Effect of health protective factors on health deficit accumulation and mortality risk in older adults in the Beijing longitudinal study of aging. J Am Geriatr Soc 2014; 62: 821–8. 24749784 10.1111/jgs.12792

[13] Lee JSW, Auyeung TW, Leung J, Kwok T, Woo J Transitions in frailty states among community-living older adults and their associated factors. J Am Med Dir Assoc. 2014; 15: 281–6. 24534517 10.1016/j.jamda.2013.12.002

[14] Ottenbacher KJ, Graham JE, Al Snih S et al. Mexican Americans and frailty: findings from the Hispanic established populations epidemiologic studies of the elderly. Am J Public Health 2009; 99: 673–9. 19197079 10.2105/AJPH.2008.143958 PMC2661466

[15] Chong MS, Tay L, Chan M et al. Prospective longitudinal study of frailty transitions in a community-dwelling cohort of older adults with cognitive impairment. BMC Geriatr 2015; 15. 10.1186/s12877-015-0174-1 PMC4696312 26715536

[16] Chamberlain AM, St Sauver JL, Jacobson DJ et al. Social and behavioural factors associated with frailty trajectories in a population-based cohort of older adults. BMJ Open 2016; 6. 10.1136/bmjopen-2016-011410 PMC4885446 27235302

[17] Collerton J, Davies K, Jagger C et al. Health and disease in 85 year olds: baseline findings from the Newcastle 85+ cohort study. BMJ 2009; 339: b4904. 20028777 10.1136/bmj.b4904 PMC2797051

[18] Kingston A, Davies K, Collerton J et al. The enduring effect of education–socioeconomic differences in disability trajectories from age 85 years in the Newcastle 85+ study. Arch Gerontol Geriatr 2015; 60: 405–11. 25747850 10.1016/j.archger.2015.02.006 PMC4407633

[19] Kingston A, Davies K, Collerton J et al. The contribution of diseases to the male-female disability-survival paradox in the very old: results from the Newcastle 85+ study. PLoS One 2014; 9: e88016. 24516578 10.1371/journal.pone.0088016 PMC3917849

[20] Jagger C, Collerton J, Davies K et al. Capability and dependency in the Newcastle 85+ cohort study. Projections of future care needs. BMC Geriatr 2011; 11: 21. 21542901 10.1186/1471-2318-11-21 PMC3097155

[21] Jackson C Multi-state models for panel data: the msm package for R 2011. J Stat Softw 2011; 38.

[22] Gill TM, Gahbauer EA, Allore HG, Han L Transitions between frailty states among community-living older persons. Arch Intern Med 2006; 166: 418–23. 16505261 10.1001/archinte.166.4.418

[23] Pollack LR, Litwack-Harrison S, Cawthon PM et al. Patterns and predictors of frailty transitions in older men: the osteoporotic fractures in men study. J Am Geriatr Soc 2017; 65: 2473–9. 28873220 10.1111/jgs.15003 PMC5681371

[24] Clegg A, Bates C, Young J, Teale E, Parry J Development and validation of an electronic frailty index using existing primary care health record data. Age Ageing 2014; 43. 10.1093/ageing/afx001 PMC6016616 28100452

[25] Stow D, Matthews FE, Barclay S et al. Evaluating frailty scores to predict mortality in older adults using data from population based electronic health records: case control study. Age Ageing 2018; 47: 564–9. 29546362 10.1093/ageing/afy022 PMC6014267

[26] Beswick AD, Rees K, Dieppe P et al. Complex interventions to improve physical function and maintain independent living in elderly people: a systematic review and meta-analysis. The Lancet 2008; 371: 725–35. 10.1016/S0140-6736(08)60342-6 PMC2262920 18313501

[27] Travers J, Romero-Ortuno R, Bailey J, Cooney M-T Delaying and reversing frailty: a systematic review of primary care interventions. Br J Gen Pract 2019; 69: e61–9. 30510094 10.3399/bjgp18X700241 PMC6301364

[28] Buckinx F, Rolland Y, Reginster J-Y, Ricour C, Petermans J, Bruyère O Burden of frailty in the elderly population: perspectives for a public health challenge. Arch Pub Health 2015; 73: 19. 25866625 10.1186/s13690-015-0068-x PMC4392630

[29] World Health Organization World report on ageing and health. Geneva 2015 [29 August 2019]; Available from: https://www.who.int/ageing/events/world-report-2015-launch/en/.

[30] Pickard S, Cluley V, Danely J et al. New horizons in frailty: the contingent, the existential and the clinical. Age Ageing 2019; 48: 466–71. 31220205 10.1093/ageing/afz032

