# Supplementary material for: Transitions between frailty states in the very old: the influence of socioeconomic status and multi-morbidity in the Newcastle 85+ cohort study
Source: Age Ageing. 2020 Apr 28;49(6):974–81. doi: 10.1093/ageing/afaa054 (PMC7583524; doi:10.1093/ageing/afaa054)

Transitions between frailty states in the very old: the influence of socioeconomic status and multi-morbidity in the Newcastle 85+ cohort study

**SUPPLEMENTARY DATA**

- **Appendix 1.** Missing pattern analysis for Fried Frailty items used.
- **Appendix 2.** Baseline sociodemographic and health characteristics of participants with and without Fried frailty score.
- **Appendix 3.** Fried frailty distribution in Newcastle 85+ Study, by sex and follow-up.
- **Appendix 4.** Number of transitions between each frailty state and death over 5 years.
- **Appendix 5.** Hazard ratios and 95% confidence intervals for socioeconomic inequalities, disease count and transitions between frailty states and death.
- **Appendix 6.** Flowchart of the Newcastle 85+ Study.
- **Appendix 7.** Fried Frailty index derived from the Newcastle 85+ Study.
- **Appendix 8.** Frailty-death multistate model with allowed transitions.

**Appendix 1. Missing pattern analysis for Fried Frailty items used.**

| **Cases in pattern** | **Exercise** | **Energy** | **BMI** | **GS** | **TUG** | **Sample without variables** |
| --- | --- | --- | --- | --- | --- | --- |
| **Baseline** | | | | | | |
| 670 |  |  |  |  |  | 670 |
| 9 |  |  |  |  | X | 679 |
| 2 |  | X |  |  | X | 711 |
| 30 |  | X |  |  |  | 700 |
| 1 | X |  |  |  |  | 671 |
| 1 | X |  |  |  | X | 681 |
| 4 | X | X |  |  | X | 717 |
| 1 |  | X |  | X | X | 714 |
| 29 |  | X | X | X | X | 812 |
| 25 |  |  | X | X | X | 748 |
| 42 |  |  | X | X |  | 714 |
| 2 |  |  |  | X |  | 672 |
| 2 |  | X | X | X |  | 746 |
| 23 | X | X | X | X | X | 845 |
| 4 | X |  | X | X | X | 754 |
| **18 months** | | | | | | |
| 497 |  |  |  |  |  | 497 |
| 24 |  |  |  |  | X | 521 |
| 2 |  | X |  |  | X | 535 |
| 12 |  | X |  |  |  | 509 |
| 2 |  | X | X | X |  | 529 |
| 18 |  |  | X | X |  | 515 |
| 32 |  |  | X | X | X | 572 |
| 1 |  |  |  | X | X | 522 |
| 23 |  | X | X | X | X | 611 |
| 93 | X | X | X | X | X | 704 |
| **36 months** | | | | | | |
| 371 |  |  |  |  |  | 371 |
| 1 |  |  |  | X |  | 372 |
| 15 |  |  | X | X |  | 387 |
| 3 |  | X | X | X |  | 402 |
| 26 |  | X | X | X | X | 483 |
| 36 |  |  | X | X | X | 438 |
| 1 |  |  |  | X | X | 387 |
| 14 |  |  |  |  | X | 385 |
| 3 |  | X |  |  | X | 400 |
| 12 |  | X |  |  |  | 383 |
| 1 |  | X |  | X | X | 403 |
| 124 | X | X | X | X | X | 607 |
| **60 months** | | | | | | |
| 255 |  |  |  |  |  | 255 |
| 6 |  |  |  |  | X | 261 |
| 2 |  |  |  | X | X | 264 |
| 1 |  |  |  | X |  | 256 |
| 11 |  |  | X | X |  | 267 |
| 30 |  |  | X | X | X | 305 |
| 20 |  | X | X | X | X | 333 |
| 95 | X | X | X | X | X | 428 |
| 1 |  | X |  | X |  | 263 |
| 6 |  | X |  |  |  | 261 |
| 1 |  | X |  |  | X | 268 |

For body mass index (BMI), energy, exercise, grip strength (GS) and Timed Up and Go (TUG), respectively, there are 14.8% (125), 10.8% (91), 3.9% (33), 15.1% (128) and 11.6% (98) of the cases missing at baseline, 23.9% (168), 18.8% (132), 13.2% (93), 24.0% (169) and 24.9% (175) at 18 months, 33.6% (204), 27.8% (169), 20.4% (124), 34.1% (207) and 33.8% (205) at 36 months, 36.4% (156), 28.7% (123), 22.2% (95), 37.4% (160) and 36.0% (154) at 60 months. Missing variables are marked with (X). Deceased participants are not included in the missing pattern analysis.

**Appendix 2.** Baseline sociodemographic and health characteristics of participants with and without Fried frailty score.

|  | **Fried**  **(n=696)** | **Non-Fried**  **(n=149)** | ***p*** | **All**  **(n=845)** |
| --- | --- | --- | --- | --- |
| **Women** | 59.5 (414) | 75.2 (112) | <0.001 | 62.2 (526) |
| **Living Status** |  |  | <0.001 |  |
| Alone | 58.0 (403) | 39.6 (59) |  | 54.7 (462) |
| Not alone | 38.9 (270) | 17.5 (26) |  | 35.1 (296) |
| Institution | 3.2 (22) | 43.0 (64) |  | 10.2 (86) |
| **Self-rated health** |  |  | 0.041 |  |
| Excellent/Very Good | 41.9 (290) | 30.3 (40) |  | 40.1 (330) |
| Good | 36.7 (254) | 42.4 (56) |  | 37.6 (310) |
| Fair/Poor | 21.4 (148) | 27.3 (36) |  | 22.3 (184) |
| **Education** |  |  | 0.007 |  |
| 0-9 years | 64.4 (447) | 64.4 (87) |  | 64.4 (534) |
| 10-11 years | 22.8 (158) | 23.0 (31) |  | 22.8 (189) |
| 12+ years | 12.8 (89) | 12.6 (17) |  | 12.8 (106) |
| **IMD** |  |  | 0.001 |  |
| More deprived (Q1) | 27.2 (189) | 14.8 (22) |  | 25.0 (211) |
| Q2+Q3 | 49.4 (344) | 51.0 (76) |  | 49.7 (420) |
| Less deprived (Q4) | 23.4 (163) | 34.2 (51) |  | 25.3 (214) |
| **SMMSE** |  |  | <0.001 |  |
| Normal (26-30) | 78.3 (544) | 38.2 (55) |  | 71.4 (599) |
| Mild (22-25) | 14.7 (102) | 22.9 (33) |  | 16.1 (135) |
| Mod (18-21) | 4.6 (32) | 10.4 (15) |  | 5.6 (47) |
| Severe (0-17) | 2.5 (17) | 28.5 (41) |  | 6.9 (58) |
| **GDS** |  |  | <0.001 |  |
| No depression | 80.9 (533) | 41.3 (45) |  | 75.3 (578) |
| Mild | 11.8 (78) | 11.9 (13) |  | 11.9 (91) |
| Severe | 5.9 (39) | 13.8 (15) |  | 7.0 (54) |
| **Disability score^1^** | 3 (1-6) | 10 (5-15) | <0.001 | 3 (1-8) |
| **Chronic diseases** |  |  | 0.001 |  |
| 0-1 | 28.2 (196) | 14.8 (22) |  | 25.8 (218) |
| 2 – 3 | 54.7 (381) | 59.7 (89) |  | 55.6 (470) |
| 4+ | 17.1 (119) | 25.5 (38) |  | 18.6 (157) |
| **Survival time** (years)**^1,2^** | 4.3 (2.4-6.4) | 2.3 (0.9-5.0) | <0.001 | 3.9 (1.8-6.2) |

Entries are percentages (%) and counts (n) unless otherwise stated. ^1^ median (interquartile range). ^2^ censored at November 2015 and, 191 and 18 participants with and without fried frailty index, respectively, were still alive. Quartiles of IMD were as follows: Q1 (25th percentile): 3-12, Q2+Q3 (25-75th percentile): 12-43, Q4 (75th percentile): 43-78. GDS, Geriatric depression scale; IMD, index of multiple deprivation; SMMSE, Standardised Mini-Mental State Examination.

**Appendix 3.** Fried frailty distribution in Newcastle 85+ Study, by sex and follow-up.

|  | **Women** | **Men** | **All** |
| --- | --- | --- | --- |
| **Baseline %(n)** | **n=414** | **n=282** | **n=696** |
| Robust | 14.7 (61) | 24.1 (68) | 18.5 (129) |
| Pre-frail | 55.6 (230) | 58.2 (164) | 56.6 (394) |
| Frail | 29.7 (123) | 17.7 (50) | 24.9 (173) |
| **18 Months %(n)** | **n=310** | **n=201** | **n=511** |
| Robust | 10.6 (35) | 19.4 (39) | 14.1 (72) |
| Pre-frail | 50.0 (159) | 59.7 (120) | 53.8 (275) |
| Frail | 39.4 (132) | 20.9 (42) | 32.1(164) |
| **36 Months %(n)** | **n=233** | **n=144** | **n=377** |
| Robust | 7.3 (17) | 14.6 (21) | 10.1 (38) |
| Pre-frail | 50.6 (118) | 56.9 (82) | 53.1 (200) |
| Frail | 42.1 (98) | 28.5 (41) | 36.9 (139) |
| **60 Months %(n)** | **n=164** | **n=94** | **n=258** |
| Robust | 3.7 (6) | 12.8 (12) | 7.0 (18) |
| Pre-frail | 51.2 (84) | 60.6 (57) | 54.7 (141) |
| Frail | 45.1 (74) | 26.6 (25) | 38.4 (99) |

**Appendix 4.** Number of transitions between each frailty state and death over 5 years.

|  | **To** | Robust | Pre-frail | Frail | Death |
| --- | --- | --- | --- | --- | --- |
| **From** |  |  |  |  |  |
| Robust |  | 91 | 111 | 9 | 19 |
| Pre-frail |  | 37 | 455 | 175 | 147 |
| Frail |  | 0 | 50 | 218 | 153 |

**Appendix 5.** Hazard ratios and 95% confidence intervals for socioeconomic inequalities, disease count and transitions between frailty states and death.

|  | **Robust to Pre-Frail** | | **Pre-Frail to Frail** | | **Pre-Frail to Robust** | | **Pre-Frail to Death** | | **Frail to Pre-Frail** | | **Frail to Death** | |
| --- | --- | --- | --- | --- | --- | --- | --- | --- | --- | --- | --- | --- |
|  | HR | 95% CI | HR | 95% CI | HR | 95% CI | HR | 95% CI | HR | 95% CI | HR | 95% CI |
| **Education** |  |  |  |  |  |  |  |  |  |  |  |  |
| 0-9 years | (ref) | | (ref) | | (ref) | | (ref) | | (ref) | | (ref) | |
| 10-11 years | 1.11 | 0.70-1.76 | 1.00 | 0.68-1.48 | 1.39 | 0.57-3.37 | 1.40 | 0.63-3.10 | 0.88 | 0.36-2.13 | 1.14 | 0.78-1.67 |
| 12+ years | 1.26 | 0.70-2.27 | 1.20 | 0.75-1.93 | 1.74 | 0.66-4.59 | 0.58 | 0.05-6.86 | 0.93 | 0.38-2.28 | 0.75 | 0.40-1.41 |
| **IMD** |  |  |  |  |  |  |  |  |  |  |  |  |
| More deprived (Q1) | 1.20 | 0.78-1.86 | 1.08 | 0.72-1.62 | 1.37 | 0.59-3.22 | 0.58 | 0.12-2.83 | 1.09 | 0.50-2.41 | 0.99 | 0.64-1.52 |
| Q2+Q3 | (ref) | | (ref) | | (ref) | | (ref) | | (ref) | | (ref) | |
| Less deprived (Q4) | 1.14 | 0.68-1.92 | 0.86 | 0.57-1.31 | 1.45 | 0.59-3.61 | 1.87 | 0.86-4.07 | 1.10 | 0.53-2.26 | 0.59 | 0.39-0.90 |
| **Disease Count** |  |  |  |  |  |  |  |  |  |  |  |  |
| 0-1 | (ref) | | (ref) | | (ref) | | (ref) | | (ref) | | (ref) | |
| 2-3 | 1.28 | 0.84-1.94 | 1.35 | 0.94-1.95 | 0.59 | 0.28-1.27 | 1.27 | 0.60-2.65 | 1.32 | 0.60-2.87 | 0.95 | 0.62-1.46 |
| 4+ | 3.79 | 1.48-9.75 | 1.65 | 1.06-2.57 | 1.31 | 0.33-5.25 | 1.11 | 0.39-3.16 | 0.55 | 0.20-1.55 | 1.39 | 0.89-2.16 |

The model was adjusted for age, sex, education, IMD and disease count. Quartiles of IMD were as follows: Q1 (25^th^ percentile): 3-12, Q2+Q3 (25-75^th^ percentile): 12-43, Q4 (75^th^ percentile): 43-78. IMD, index of multiple deprivation.

**Appendix 6.** Flowchart of the Newcastle 85+ Study.

**
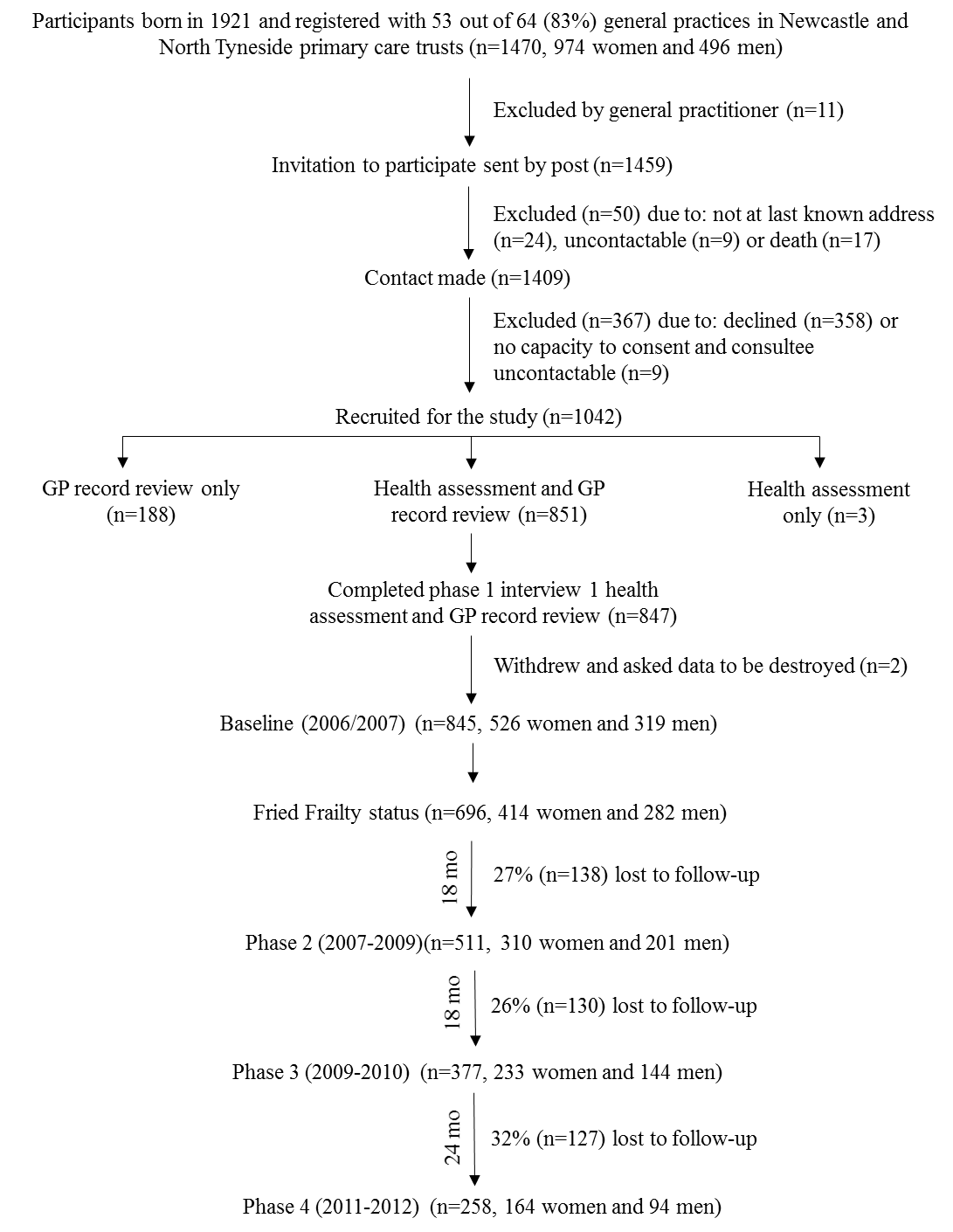
**

**Appendix 7**. Fried Frailty index derived from the Newcastle 85+ Study.


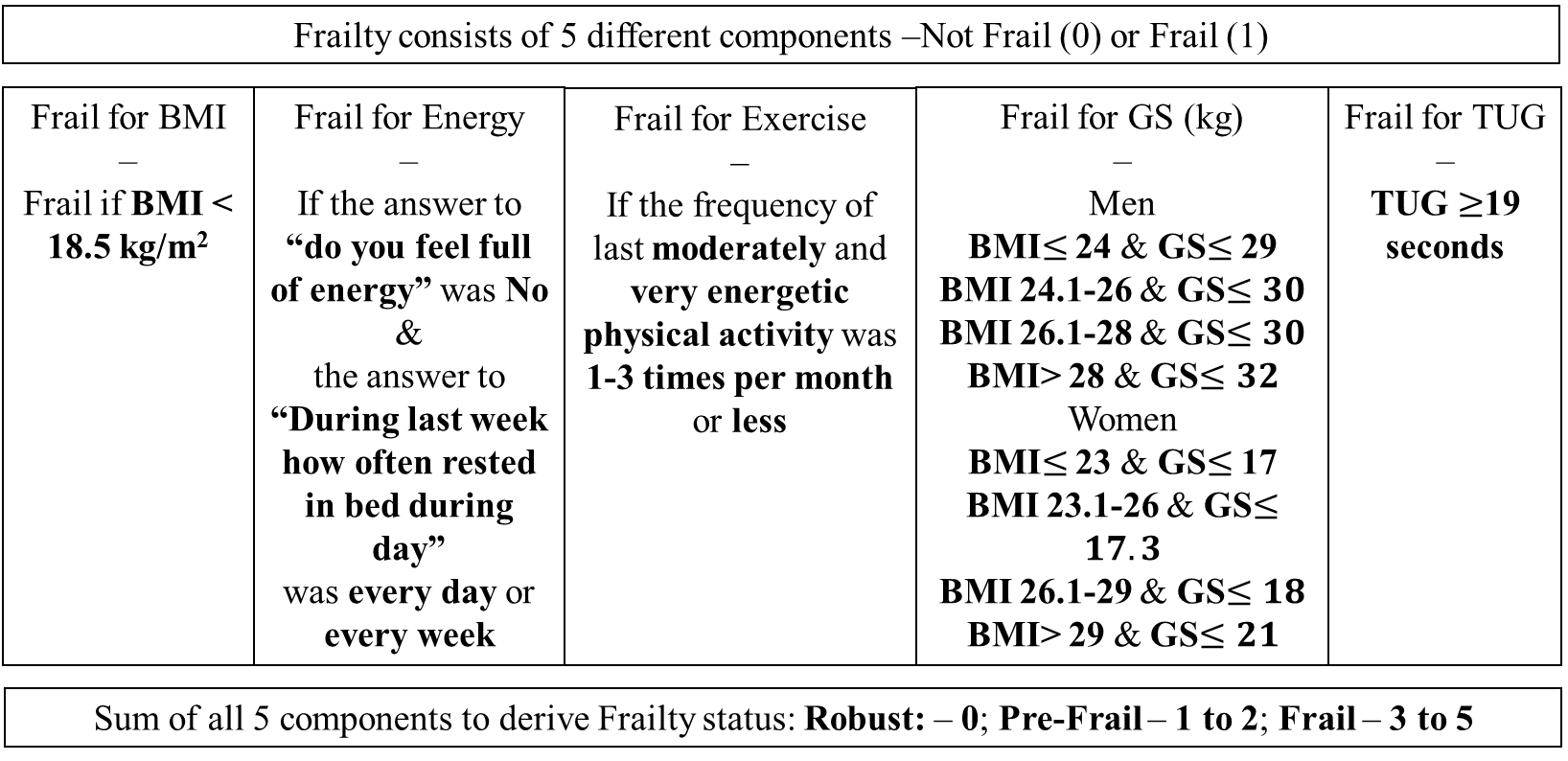
 Scores imputed as Robust if total score was 0 with no item missing, as Pre-Frail if total score was 1 with one item missing, and as Frail if total score was 3 or 4 with 1 or 2 items missing. BMI, body mass index; GS, grip strength; TUG, Timed Up and Go.

**Appendix 8.** Frailty-death multistate model with allowed transitions.


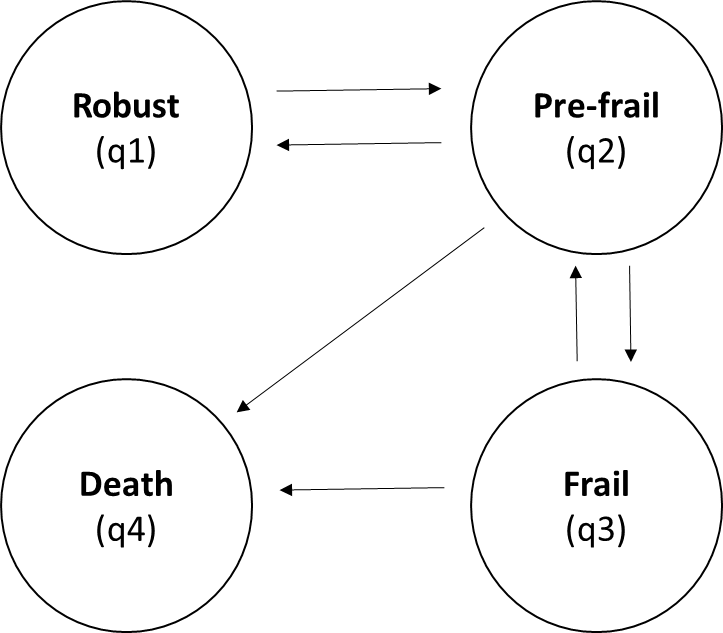

Supplement: File002_afaa054 [file file002_afaa054.docx]
